# More women than ever are entering MD-PhD programs. What lies ahead for them?

**DOI:** 10.1172/jci.insight.184715

**Published:** 2024-11-22

**Authors:** Lawrence F. Brass, Myles H. Akabas

**Affiliations:** 1 University of Pennsylvania Medical Scientist Training Program, Philadelphia, Pennsylvania, USA.; 2 Albert Einstein College of Medicine Medical Scientist Training Program, New York, New York, USA.

**Keywords:** Aging, Adaptive immunity

## Abstract

The earliest MD-PhD programs were small and enrolled mostly men. Here, we show that since 2014 there has been a steady increase in the number of women in MD-PhD programs, the number of women reaching parity with men in 2023. This change was due to an increase in female applicants, a decrease in male applicants, and an increase in the acceptance rate for women, which had previously been lower than that for men. Data from the National MD-PhD Program Outcomes Study show that training duration has been similar for men and women, as have most choices of medical specialties and workplaces. However, women were less likely to have full-time faculty appointments, fewer had NIH grants, and those in the most recent graduation cohort at the time of the survey reported spending less time on research than men. Previously cited reasons for these differences include disproportionate childcare responsibilities, a paucity of role models, insufficient recognition, and gender bias. Institutions can and should address these obstacles, but training programs can help by preparing their graduates to succeed despite the systemic obstacles. The alternative is a persistent gender gap in the physician-scientist workforce, lost opportunities to benefit from diverse perspectives, and a diminished impact of valuable training resources.

## Abstract 2

Women are underrepresented in the physician-scientist workforce. Here we document the recent surge in female applicants to MD/PhD programs and consider the obstacles they will face.

## Introduction

MD-PhD programs were first established in the 1950s to attract and train future physician-scientists by combining medical school with research training in graduate school. The earliest programs were small, and most of their trainees were men (1, 2). Over the six decades since, the number of programs and the number of trainees has risen considerably. However, for many years the sex imbalance in MD-PhD program trainees persisted, even as the number of women in medical school and biomedical PhD programs reached and then exceeded parity with that of men.

Here, we have used publicly available data provided by the Association of American Medical Colleges (AAMC) to examine recent trends in the number of women and men applying to and entering US MD-PhD programs. We then combined those numbers with comparable information on gender trends in medical school applicants, performance metrics from the National MD-PhD Program Outcomes Study (1–3), previously reported data on the prevalence and causes of postbaccalaureate gaps before medical school (4), and data from our own institutions (the University of Pennsylvania and the Albert Einstein College of Medicine) on the publication records of MD-PhD program students.

The results show that over the 5-year period from 2016 to 2021 there was a steady increase in the number of women entering MD-PhD programs, a trend that was also noted in a study on Harvard and MIT’s MD-PhD program published in 2020 (5). The increase in the number of women was accompanied by a decline in the number of men. At present, women are joining MD-PhD programs at approximately the same rate as men. Total enrollment, which is a lagging indicator, is approaching parity, as presumably will the number of graduates each year, assuming equally low attrition rates for women and men. The physician-scientist workforce in the US includes both MDs and MD-PhDs as well as a smaller number of DOs and DO/PhDs. Historically, men in this workforce have greatly outnumbered women. What will be the impact of increasing numbers of women entering into MD-PhD programs? Is the gender gap in the physician-scientist workforce at last on course to close?

Recent data are not entirely encouraging. Although, among MD-PhD students, women are as likely as men to have spent one or more years doing research after college before matriculating into medical school (4), outcomes data for performance before and after graduation from MD-PhD programs give a more nuanced picture. Women who have graduated from MD-PhD programs so far have taken approximately the same amount of time as men to complete the program and, with several notable exceptions, selected similar clinical fields for residency training after graduation (1). However, in surveys conducted in 2015 for the National MD-PhD Outcomes Study (1, 3), women who graduated between 2000 and 2014 and had completed postgraduate training reported devoting less of their professional time to research, and female alumni in general were a bit less likely to have chosen careers in academia (66% for men vs. 63% for women) and held fewer NIH grants (1, 3). The difference in the fraction of men and women graduates working in academia was even greater (men, 60% vs. women, 52%) in a search of the AAMC Faculty Roster Database that included graduates who did not respond to the survey as well as those who did respond 60% vs. 52%) (1, 3). In this brief report, we examine the data behind this summary and consider some of the reasons for the differences that we and others have observed.

## Results

### Matriculation data.

**Figure 1:**
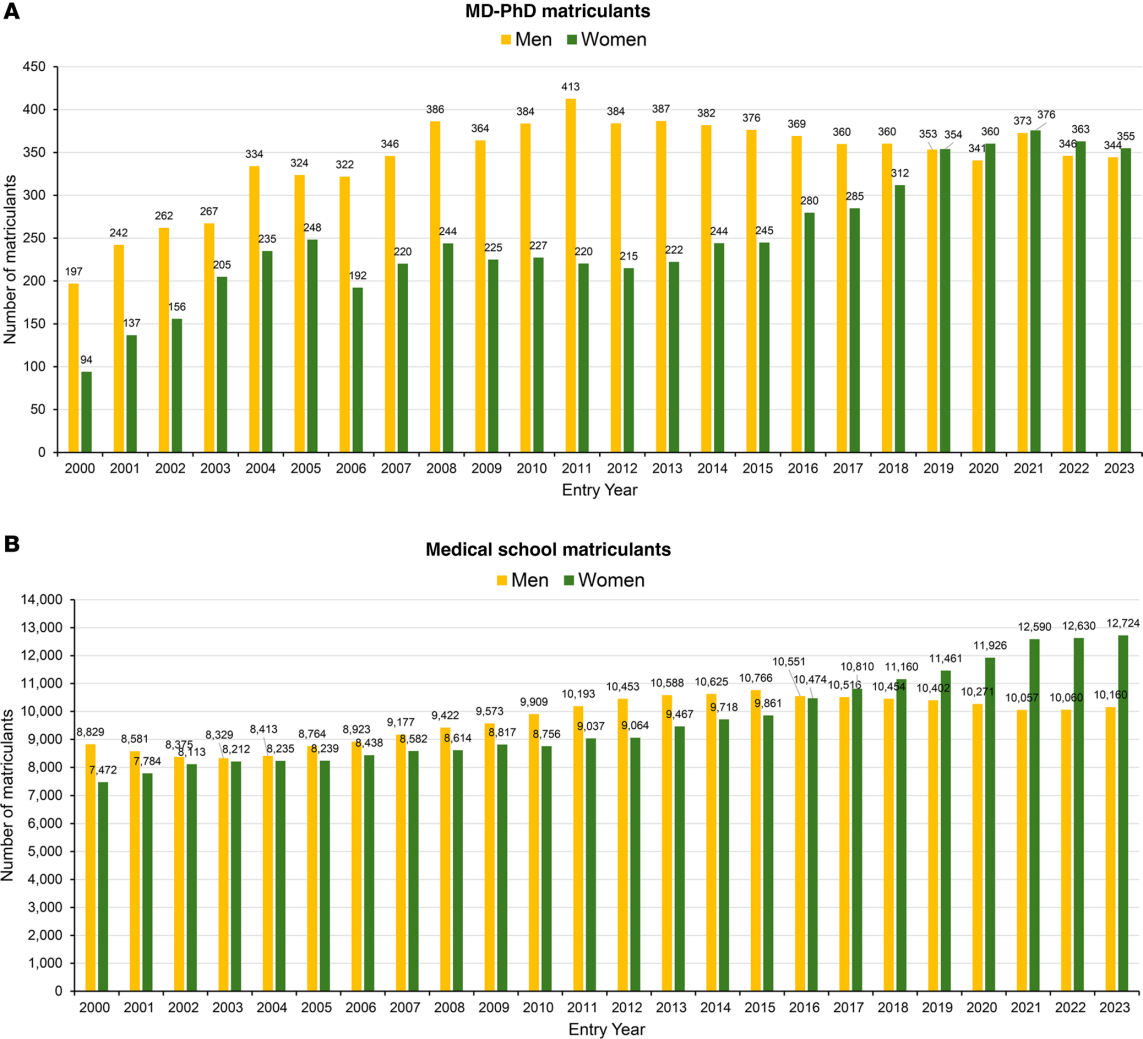
Matriculation into MD-PhD programs and medical school by entry year. (**A**) MD-PhD programs. (**B**) Medical school. Medical school matriculants are approximately 97% MD and 3% MD-PhD. Datasets are publicly available from the Association of American Medical Colleges (AAMC) (33).

Figure 1A shows matriculation into MD-PhD programs, beginning with cohorts who started training in 2000. In that year, there were 291 entering MD-PhD candidates, 32% of whom were women. In 2023, more than twice as many MD-PhD candidates (699 candidates) entered medical school; 51% were women. The surge in the number of women began in 2013, peaking at 376 in the class entering in 2021. The number of men entering MD-PhD programs reached a high of 413 in 2011 (when the entering class was 65% men and 35% women) and drifted downward to 341 in 2020 (when the entering class was 49% men and 51% women).

Medical school admissions show a similar trend generally: an increase in overall numbers and a rising percentage of the candidates who are women (Figure 1B). Note that “medical school matriculants” includes MD and MD-PhD candidates, but this group comprises approximately 97% MD candidates. In the class entering in 2023, there were 22,282 MD candidates and 699 MD-PhD candidates (3.1%).

### Enrollment.

**Figure 2:**
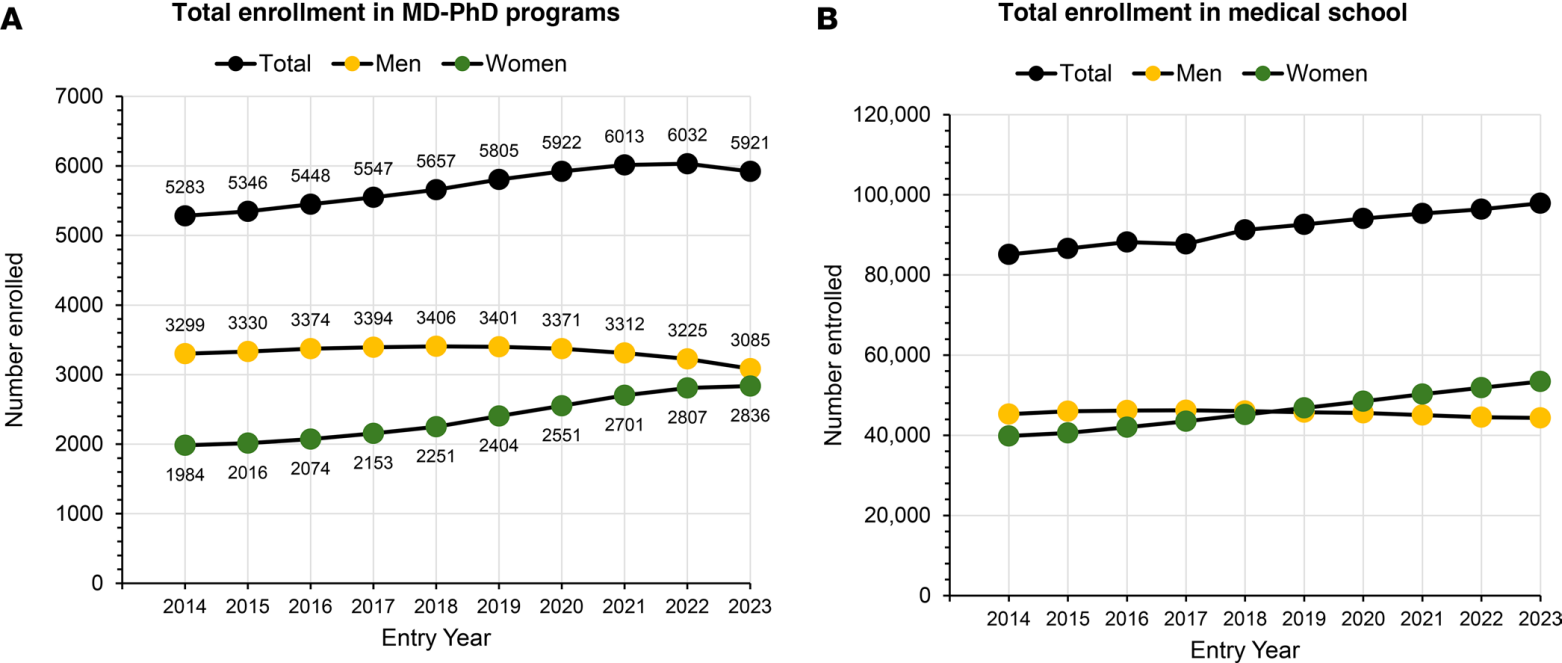
Total enrollment in MD/PhD programs and medical school by entry year. (**A**) MD-PhD programs. (**B**) Medical school. Datasets are publicly available from the Association of American Medical Colleges (AAMC) (33).

Overall enrollment in MD-PhD programs and medical schools is shown in Figure 2. Changes in total enrollment lag changes in matriculation. In the decade starting in 2014, enrollment in MD-PhD programs increased from 5,283 to 6,032, a 14% increase (Figure 2A). In 2014, 38% of the 5,283 students enrolled in MD-PhD programs were women; 62% were men. In the 2023 entry year, 48% of 5,921 students enrolled in MD-PhD programs were women; 52% were men. Parity is expected within the next several years. Enrollment in medical school reached parity in 2018 and has been exceeded in the years since then (Figure 2B). In the academic year beginning 2023, 55% of enrolled medical students (53,422 of 97,903) were women.

### Applicants.

**Figure 3:**
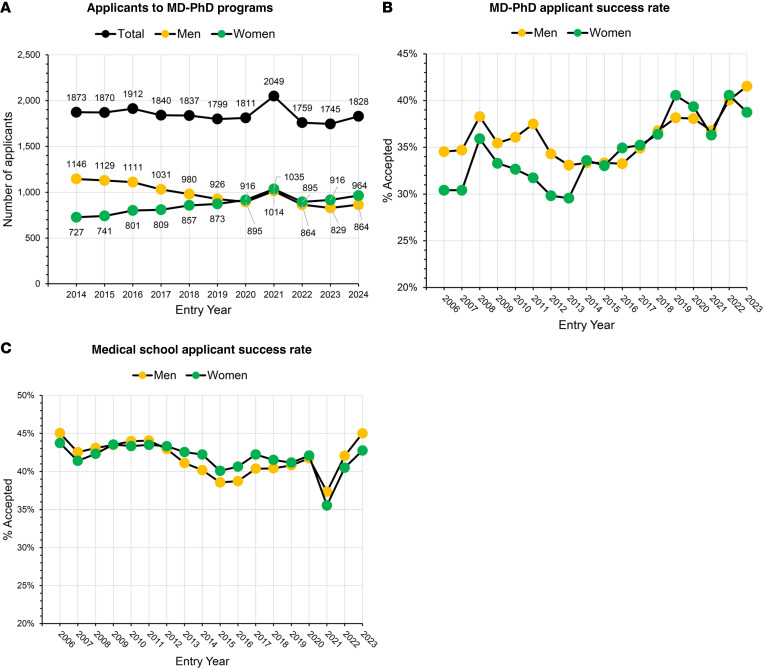
Applicants and applicant success. (**A**) The number of applicants to MD-PhD programs (2014-2024). See Supplemental Table 1 (supplemental material available online with this article; https:// doi.org/10.1172/jci.insight.184715DS1). (**B**) Applicant success rate for MD-PhD programs (2006–2023). The numbers shown were derived by dividing the number of matriculants from each of those years (see Figure 1A) by the number of applicants in those same years. See Supplemental Table 2. (**C**) The success rate for applicants to medical school was derived by dividing the number of matriculants by the number of applicants. See Supplemental Table 3. Note: The AAMC routinely updates information about applicants to MD-PhD programs, causing the numbers for past years to change over time. To prepare the graphs shown in **A** and **B**, a unified applicant dataset from 2006 to 2024 was constructed as follows. Applicant data from 2006 to 2009 are from saved versions of AAMC Data Table 32 for those years. Data Table 32 is no longer posted online but is available by request from the AAMC data unit, as are applicant numbers from 2021 to 2024. Applicant numbers 2010–2020 are from ref. 4. The complete dataset constructed in this manner is included in Supplemental Tables 1 and 2.

The number of applicants to MD-PhD programs each year has held steady since 2014 (Figure 3A). However, this steadiness hides an increase in the number of women applying and a concomitant decline in the number of men applying each year. Acceptance rate data show that from 2006 until 2013, women were consistently accepted into MD-PhD programs at a lower rate than men (Figure 3B). Parity was achieved in 2014 and has continued. Thus, the upward surge in the number of women annually joining MD-PhD programs since 2013 reflects an increase in the number of women applying and an increase in their rate of acceptance by programs. Notably, the medical school acceptance rate during this same period was generally higher than that for MD-PhD programs. There was also no consistent difference in acceptance rate between men and women (Figure 3C).

## Discussion

MD-PhD programs are not the only available path to becoming a physician-scientist, but holders of both doctorates represent a share of physicians who hold NIH grants that is disproportionate to their approximately 3% prevalence among medical school entrants (6). In fiscal years 2014–2023, dual-degree holders held two-thirds of the approximately 96,000 NIH research project grants held by physicians, rising from 61% in 2014 to 70% in 2023 (7). For many years, discussions about admissions to MD-PhD programs included the observation that the percentage of women entering medical school in the US and elsewhere exceeded 50%, as did the number of women in biomedical PhD programs. However, when medical school and graduate school were combined in an integrated MD-PhD program, the number of men greatly exceeded the number of women. The reasons for this difference were never determined.

Since greater diversity is one of the goals of the physician-scientist training community, we have been pleased to note the surge in the number of women entering MD-PhD programs since 2013. Parity among applicants has been achieved, and enrollment, which is a lagging indicator, is very close. However, increases in total enrollment have not been accompanied by increases in the number of applicants, in part because the number of men applying each year has declined. To some extent the increase in women and the decrease in men in MD-PhD programs reflects a national trend in college attendance: more women than men are graduating from college. In 2022, 48% of women from 18 to 24 years of age were in college compared with only 39% of men (8). Whatever the driver for these changes, an increase in the number of women entering MD-PhD programs is good news. It would be even better news if the number of men had at least held steady.

*What lies ahead?* Based on past experience, most people who begin an MD-PhD program will graduate with both doctorates (9). What lies ahead for the women who are in MD-PhD programs today? Will they meet their training goals, achieving careers that take advantage of their research training as well as their clinical training? Put differently, will the large (and growing) national investment in women in MD-PhD programs continue to provide a good return? Historical data from the National MD-PhD Program Outcomes study do show that female graduates of MD-PhD programs can succeed as physician-scientists. In that study of over 10,000 graduates, total training duration, which includes time to graduation as well as time to first independent position, was similar for men and women. So were their choices of most medical specialties for postgraduate training, a decision that has long-term implications for their ability to do research if they are in academia (2). Regardless of sex, most participants in the outcomes study reported that they are working in academia, research institutes, federal agencies, or industry. The National Outcomes study included five decades of graduates. If anything, the career preferences stated by MD-PhD program alumni who were still in residencies at the time of the 2015 survey suggest that an even greater percentage of them will have careers in academia.

### Differences between male and female graduates.

These similarities between women and men were, however, accompanied by several differences. As noted in the introduction, based on their survey responses and a search of the AAMC Faculty Roster Database, women were less likely than men to have full-time faculty appointments, fewer had NIH grants, and fewer reported success in moving from a mentored to an independent NIH award (1, 3). Perhaps accounting for some of these differences, women in the 2000–2014 graduation cohort (the most recent cohort at the time of the survey) who held full-time academic appointments self-reported spending less time on research than men (1, 3). Other differences exist as well. A single-institution study from the University of Wisconsin’s MD-PhD program noted that women in the program asked fewer questions to seminar speakers than their male peers (10). They also published fewer articles than the men in their program. Although this latter difference did not reach statistical significance, it prompted us to look at publication records from the MD-PhD programs that we direct at the University of Pennsylvania and the Albert Einstein College of Medicine. The average number of graduate school–related publications for graduates of the University of Pennsylvania from 2007 to 2023 was 6.20 ± 3.98 for men (*n* = 207, mean ± SD) and 4.72 ± 3.06 for women (*n* = 106, mean ± SD) (*P* = 0.000861 by unpaired *t* test). The average number of publications for Einstein graduates from 2007 to 2023 was 5.82 ± 3.52 for men (*n* = 131, mean ± SD) and 4.33 ± 2.26 for women (*n* = 86, mean ± SD) (*P* = 0.000188).

Longer-term data also indicate that on average women in the physician-scientist workforce and academia are not thriving as well as men. While women and men in MD-PhD programs are equally likely to receive NIH F30 training awards and NIH-mentored research (K) awards, women who do receive K awards are less likely to subsequently receive NIH research program grant (R) funding (11). In 2023 only one-third of NIH research program grants were awarded to women and the award sizes were on average smaller (11–14). Women were also more likely than men to leave the NIH funding pool after a single unsuccessful proposal (13), and those who remain are underrepresented in the lists of high-profile awards (15). However, among those who persist, the success rate for NIH research project grant applications for men and women is similar (14). In a national study, women in academia were less likely than men to be promoted from assistant to associate professor and even less likely to be promoted to full professor (16).

### Reasons behind the differences.

Sadly, the reasons for these differences have been reviewed and discussed at conferences and in the biomedical literature for years without substantial improvement. The problem is not unique to the US (17). The National MD-PhD Outcomes Study alumni survey documented achievements and career paths. It did not address reasons. However, it is not hard to find them. Women in the US bear a disproportionate share of responsibilities for child and elder care, responsibilities that can reduce the time available for demanding careers. Graduates of MD-PhD programs almost always go on to postgraduate clinical training and maintain active clinical credentials. Most of those in academia work in clinical rather than basic science departments and, as a result, have clinical service obligations that may or may not be directly congruent with their research interests (3). Providing clinical care for patients can be an all-consuming career path, but it can also provide greater flexibility and a greater opportunity for part-time work. There are also gender differences in mentorship, sponsorship, and resource allocation as well as a paucity of female role models (12). Startup packages for women can be lower than for men (18). Publications led by women are cited less frequently, which can lead to less recognition (19–22). Finally, the working environment at universities can produce problems for women that are less likely to be faced by men: half of the women in schools of medicine reported discrimination or sexual harassment in a 2018 National Academies report (23).

These differences by sex add up to an additional burden in an already challenging career. To what extent does this burden push women in their 30s and 40s off a career path that they chose in their late teens and early 20s? Will the current underrepresentation of women in the physician-scientist workforce in academia persist or, given sufficient time, will the surge of women coming out of MD-PhD programs close the gap?

### Why we should care.

Finally, should we care if female graduates of MD-PhD programs prove to be collectively less successful as physician-scientists than their male peers? There are reasons why we should. One is the evidence that gender diversity in research teams has a positive effect, both by increasing metrics of impact and by drawing attention to a wider range of investigation (24–26). Although the evidence supporting this conclusion for the physician-scientist workforce remains comparatively limited, the conclusions parallel those from studies in fields such as management and business (27).

A second reason for caring about the careers of women who wish to become physician-scientists is the sizable investment that each MD-PhD student represents. The NIH and medical schools invest large sums in the training of MD-PhD candidates, covering their tuition and stipends and supporting program costs for faculty and staff time, activities, and infrastructure. This does not mean that there are not short-term benefits to each medical center for doing this: MD-PhD students join faculty-led research teams and through their efforts help to attract research dollars. Longer term, MD-PhD alumni become faculty members, but arguably their intended impact is reduced if they become academic clinicians rather than productive physician-scientists. Devoting four years to biomedical research training is not a priority for a clinical career however much it may expand a clinician’s perspectives on patient management.

A third reason is entirely practical: NIH research institutes, especially the National Institute of General Medical Sciences (NIGMS), provide millions of dollars to fund MD-PhD programs, and in return they rightly expect that many of our graduates will become research-focused physician-scientists. Any assessment of the success of a medical scientist training program has to consider the long-term career success of all of its graduates. Most students who begin an MD-PhD complete it (9). Success lies, therefore, not in graduation rates but in their eventual choice of workplace, the time that they can devote to research and discovery, and the impact that the graduates have had through their individual and team efforts in academia, industry, and government. Ignoring what happens to female graduates is, therefore, not an option. MD-PhD programs have to care about the success of each individual.

### Interventions.

Given the challenges of the career, what can be done to improve outcomes? Fortunately, there are examples of interventions that had a positive effect, even if they are not necessarily focused on physician-scientist training and trainees. Female peer mentors have been shown to help female engineering students improve their experience in engineering, aspire to pursue postgraduate engineering degrees, and achieve emotional well-being. In that study, female students who were assigned female mentors did better than those with male mentors or no mentor (28). A study performed at Yale’s MD-PhD program showed that interventions to encourage and support F30 and F31 applications improved the application rates and award success for female as well as male students (29). Analysis of 10 years of outcomes from the Sallie Rosen Kaplan postdoctoral fellowship program at the National Cancer Institute showed increased self-confidence, improvements in time management and work/life balance, and enhanced goal setting and attainment of skills among the female fellows (30). Finally, the presence of women among conference organizers has been shown to increase the number of women invited to be speakers at conferences, indirectly increasing the presence of female as well as male role models for trainees attending the conference (31).

In addition to these examples, a number of thoughtful commentaries have offered actionable solutions to bolster the success of women who have already joined the physician-scientist workforce (10, 12, 13, 17, 32). Those include achieving salary and start-up package equity, balancing calls for uncompensated service and teaching time to be sure that women do not bear a disproportionate burden at work, hiring and rewarding more female role models, providing high-quality on-campus childcare, limiting meetings in the evenings and weekends, and including more women on promotion and tenure committees.

We will close by saying that in our opinion the entry of more women into MD-PhD programs is an event to be celebrated, but it calls on all of us who care about the physician-scientist community to do everything we can to ensure their success. The alternative is a persistent gender gap in the physician-scientist workforce, lost opportunities to benefit from diverse perspectives, and a diminished impact of valuable training resources.

## Methods

### Sex as a biological variable.

This study is about the changing demographics of students in MD-PhD programs. As a result it includes data on both women and men who have applied to and graduated from MD-PhD programs.

### Application, matriculation, and enrollment data.

Except where noted, application, matriculation, and enrollment data for MD-PhD and MD students in US medical schools are from publicly posted datasets provided by the AAMC (33).

### Data on research effort and research grants.

Data on research effort and research grants for MD-PhD program alumni are from the 2015 National MD-PhD Program Outcomes Survey and AAMC data on the 6,786 individuals who completed the survey (3). Eighty MD-PhD programs, including all but 1 of the 45 programs that received NIGMS Medical Scientist Training Program (MSTP) grants in 2015, participated in that study. The programs identified 10,591 alumni and provided valid email addresses for 8,944 (84%) to the AAMC data unit. Each person received an email from the current director of the program from which they graduated informing them that the program was participating in a national outcomes study and that they would receive an email on a specified date from the AAMC with an individualized, active URL link to the online survey. Survey responses from 6,786 graduates (76% of 8,944) were collected on an AAMC server using Verint software. Survey response rates were the same for men (64%) and women (66%). Data for gender and the years of matriculation and graduation were obtained from AAMC databases for each survey respondent as described previously (3).The AAMC Institutional Review Board approved the survey and the data collection and analysis processes. The authors have signed a data-sharing agreement with AAMC.

### Data on postbaccalaureate gaps.

Data on postbaccalaureate gaps between college and medical school were provided by the AAMC and students who were enrolled in MD-PhD programs in 2021 (4). Survey information about the reasons for having gaps before medical school were obtained as previously described (4).

### Study approval.

Analysis of data for graduates from the Albert Einstein College of Medicine MSTP and the University of Pennsylvania MSTP was performed under the auspices of Albert Einstein College of Medicine IRB protocol 2021-12869 and was deemed to be exempt from federal human research regulations (45 CFR 46).

### Data availability.

Values for all data points in graphs are reported in the Supporting Data Values file.

## Author contributions

This project was conceived jointly by the two authors. Both LFB and MHA wrote the manuscript. LFB and MHA have collaborated on numerous projects, and they alternate who appears as the first and last author their papers.

## Supplementary Material

Supplemental data

Supporting data values

## Version 1 10/15/2024

In-Press Preview

## Version 2 11/22/2024

Electronic publication
