# Supplementary material for: More women than ever are entering MD-PhD programs. What lies ahead for them?
Source: JCI Insight. 2024 Nov 22;9(22):e184715. doi: 10.1172/jci.insight.184715 (PMC11601917; doi:10.1172/jci.insight.184715)
Supplement: Supplemental data [file jciinsight-9-184715-s228.pdf]

Supplementary materials for

More women than ever are entering MD-PhD programs. What lies ahead for them?

Lawrence F. Brass and Myles H. Akabas

Lawrence F. Brass, MD PhD
Professor of Medicine and Pharmacology
Director, University of Pennsylvania Medical Scientist Training Program
Room 815 BRB-II/III
421 Curie Blvd
Philadelphia, PA 19104
215-573-3540

Myles H. Akabas, MD PhD
Professor of Neuroscience and Medicine
Director, Albert Einstein College of Medicine Medical Scientist Training Program
1300 Morris Park Ave
Bronx, NY 10461
718-430-3360

Table S1. Applicants to MD/PhD programs

Entry year	Total	Men	Women
2014	1873	1146	727
2015	1870	1129	741
2016	1912	1111	801
2017	1840	1031	809
2018	1837	980	857
2019	1799	926	873
2020	1811	895	916
2021	2049	1014	1035
2022	1759	864	895
2023	1745	829	916
2024	1828	864	964

Table S2. MD/PhD applicant success rate

Entry year	**Men Applicants**	**Women Applicants**	**Men Matriculants**	**Women Matriculants**	**Rate for men**	**Rate for women**
2006	932	632	322	192	35%	30%
2007	997	724	346	220	35%	30%
2008	1009	679	386	244	38%	36%
2009	1027	676	364	225	35%	33%
2010	1064	696	384	227	36%	33%
2011	1101	694	413	220	37%	32%
2012	1120	721	384	215	34%	30%
2013	1169	752	387	222	33%	30%
2014	1146	727	382	244	33%	34%
2015	1129	741	376	245	33%	33%
2016	1111	801	369	280	33%	35%
2017	1031	809	360	285	35%	35%
2018	980	857	360	312	37%	36%
2019	926	873	353	354	38%	41%
2020	895	916	341	360	38%	39%
2021	1014	1035	373	376	37%	36%
2022	864	895	346	363	40%	41%
2023	829	916	344	355	42%	39%

Table S3. Medical school applicant success rate

Entry year	Men Applicants	Women Applicants	Men Matriculants	Women Matriculants	Acceptance rate for men	Acceptance rate for women
2006	19,815	19,293	8,923	8,438	45%	44%
2007	21,580	20,735	9,177	8,582	43%	41%
2008	21,870	20,360	9,422	8,614	43%	42%
2009	22,013	20,252	9,573	8,817	43%	44%
2010	22,533	20,207	9,909	8,756	44%	43%
2011	23,135	20,780	10,193	9,037	44%	43%
2012	24,338	20,922	10,453	9,064	43%	43%
2013	25,760	22,250	10,588	9,467	41%	43%
2014	26,455	23,019	10,625	9,718	40%	42%
2015	27,927	24,608	10,766	9,861	39%	40%
2016	27,250	25,779	10,551	10,474	39%	41%
2017	26,058	25,600	10,516	10,810	40%	42%
2018	25,875	26,882	10,454	11,160	40%	42%
2019	25,494	27,847	10,402	11,461	41%	41%
2020	24,620	28,343	10,271	11,926	42%	42%
2021	26,948	35,438	10,057	12,590	37%	36%
2022	23,924	31,191	10,060	12,630	42%	40%
2023	22,574	29,763	10,160	12,724	45%	43%
